# Compound intervention affects pathology in a novel humanized APP Knock‐In mouse model (APP^SAA^), strengthening its relevance as a tool in preclinical research

**DOI:** 10.1002/alz.088809

**Published:** 2025-01-09

**Authors:** Wannes Dejonckheere, Sofie Carmans, Tom Cornelissen

**Affiliations:** ^1^ reMYND, Leuven Belgium

## Abstract

**Background:**

To improve clinical translatability of non‐clinical in‐vivo Alzheimer’s disease (AD) models, a humanized APP knock‐in mouse model (APP^SAA^) was recently created (Xia, D. et al., 2022). The genetic modifications lead to increased Aβ42/40 ratios in AD relevant tissues, resulting in an age‐dependent amyloid deposition. Here we assess the value of this model for non‐clinical efficacy studies of experimental treatments with diverse mechanisms of action to facilitate the development of novel AD therapeutics.

**Method:**

APP^SAA^ mice and WT controls were aged and sacrificed at various time points. In addition, APP^SAA^ mice were treated for 3 months from the age of 3 months onward with an NLRP3‐inhibitor, an in‐house developed calcium homeostasis modulator or vehicle. The major pathological hallmarks were investigated with biochemical and immunohistological assays. Additionally a preliminary screen for AD biomarkers was conducted in CSF samples. Since APP^SAA^ mice lack an obvious behavioral phenotype, synaptic plasticity was investigated to serve as a functional readout.

**Result:**

Cortical Aβ40, Aβ42 and pyroglutamate modified Aβ42 (N3pE‐42) levels were shown to be affected with age. In addition, cortical amyloid plaques, surrounded by activated microglia, were shown to be present already at an age of 3 months, albeit at a low level. With age, the number of cortical plaques increased. In addition, we were able to confirm the presence of endogenous phospho‐Tau positive dystrophic neurites within these plaques, clearly demonstrating the translational relevance of this model. Interestingly, several of the observed hallmarks were counteracted by compound intervention, including phospho‐Tau pathology. Finally, we show that several CNS biomarkers can be detected in mouse CSF.

**Conclusion:**

These data lead us to conclude that this knock‐in model is a broadly applicable tool to investigate efficacy of disease‐modifying drugs with diverse mechanisms of action even without directly targeting protein aggregates.

